# Agreeableness and Subjective Well-Being: The Mediating Role of Perceived Social Support as a Coping-Relevant Resource and the Moderating Effect of Family Income

**DOI:** 10.3390/bs16010038

**Published:** 2025-12-24

**Authors:** Xuefei Deng, Jianwen Chen

**Affiliations:** School of Education, Huazhong University of Science and Technology, Wuhan 430070, China; phifaye@hust.edu.cn

**Keywords:** agreeableness, subjective well-being, perceived social support, family yearly income, moderated mediation

## Abstract

This study investigates the role of Agreeableness as a personality trait in promoting psychological well-being, with a specific focus on the potential mediating mechanism of social support, and how this pathway is influenced by family’s income. 3206 college students from China’s universities were recruited from Internet, randomly. Subjects were demanded to complete the Agreeableness Subscale of Chinese Big Five Inventory Brief version (CBF-PI-B), the Chinese Campbell Index of Well-Being (Campbell IWB), the Chinese Perceived Social Support Scale (PSSS) and demographic variables. The results, analyzed using a moderated mediation procedure, confirmed that perceived social support mediates the relationship between Agreeableness and subjective well-being. Furthermore, family yearly income was found to significantly moderate the first stage of this mediation pathway. Specifically, the positive associative effect of Agreeableness on perceived social support was stronger for individuals with lower annual family income. This result suggests that, for those with fewer economic resources, a prosocial and agreeable disposition is a particularly critical asset for building the social support networks that subsequently enhance well-being. The findings highlight the complex interplay between personality and socioeconomic context, indicating that social support serves as a healthy coping mechanism, the utility of which is conditionally shaped by an individual’s financial circumstances.

## 1. Introduction

### Literature Review

Subjective well-being (SWB) has long been a central focus in the field of psychology, attracting the attention of numerous scholars. As a global evaluation of an individual’s quality of life based on their self-defined standards, it serves as a key comprehensive psychological indicator for assessing personal life quality in psychological research (Diener, 1984). Generally, SWB consists of a person’s cognitive and affective evaluation of his or her life (Diener et al., 1999).

Over the past few decades, extensive research has explored the factors influencing SWB. Among them, personality traits have been found to play a significant role. The Five Factor Model of personality, which includes Neuroticism, Extraversion, Openness to Experience, Conscientiousness, and Agreeableness, has been widely used in the study of the relationship between personality and SWB. Previous research has clearly demonstrated that Neuroticism and Extraversion have relatively consistent and significant impacts on SWB. For example, individuals high in Neuroticism are more likely to experience negative emotions and have lower levels of life satisfaction, while those high in Extraversion tend to have more positive emotions and higher SWB (Busseri & Erb, 2024).

However, the relationship between Agreeableness and SWB is still a topic of debate. Agreeableness refers to an individual’s tendency to be kind, cooperative, empathetic, and considerate in interpersonal relationships (John & Srivastava, 1999). Given that human beings are social animals and interpersonal relationships are an important part of life, it is reasonable to assume that Agreeableness, which is closely related to interpersonal interactions, should have an impact on SWB. But the existing research results are inconsistent, with some studies suggesting a positive correlation between Agreeableness and SWB (Sheldon et al., 1997; DeNeve & Cooper, 1998), while others indicating a significant negative correlation (Burns & Machin, 2010). Chinese scholars, such as X. Zhang and Zheng (2005) and Tang et al. (2010) also have the similar findings. This inconsistent situation has led to a lack of a unified understanding of the nature of the relationship between Agreeableness and SWB, leaving room for further exploration and research. Therefore, it is necessary to review and analyze comprehensively the existing research to clarify this complex relationship.

Beyond personality, perceived social support—defined as individuals’ subjective appraisals of available support resources, including emotional, instrumental, and informational assistance (Lakey & Orehek, 2011)—has been identified as a critical predictor of SWB across diverse populations. Unlike objective or enacted support, perceived social support reflects “psychological reality”—a factor deemed more influential for well-being than external support availability (Coyne & Downey, 1991). Some scholars, Wilmot and Ones (2022), Zhao et al. (2019), found that higher perceived social support was robustly associated with greater life satisfaction and lower negative affect among young adults, even after controlling for objective socioeconomic status. Similarly, in a study of older adults, Newson and Schulz (1996) confirmed that perceived emotional support exerted a stronger predictive effect on SWB than instrumental or informational support, aligning with earlier findings that emotional support buffers stress and fosters a sense of belonging. This pattern extends to vulnerable groups: D. Zhang et al. (2021) reported that among Chinese college students, perceived social support mediated the relationship between prosocial tendencies and SWB, emphasizing its role as a bridging mechanism between individual traits and well-being outcomes.

Compounding this complexity, individual differences in personality, particularly Agreeableness, shape the perception of social support. Agreeableness has been consistently linked to more positive social interactions and higher perceived support across lifespan samples. S. J. Branje et al. (2004) found that Agreeableness correlated most strongly with perceived social support among adolescents, a relationship attributed to highly agreeable individuals’ tendency to engage in prosocial behaviors and build trust-based relationships. This association is not unidirectional: S. J. T. Branje et al. (2005) noted that agreeable individuals not only perceive more support but are also viewed as more supportive by others, creating a reciprocal cycle that reinforces social connection. Recent research further clarifies the cognitive mechanisms underlying this link: Lakey et al. (2021) proposed that agreeable individuals’ enhanced empathy leads them to attribute positive intentions to others, thereby increasing their likelihood of perceiving available support—a process consistent with Beck’s cognitive theory, which posits that self-other schemas influence subjective appraisals (Beck, 1976). Cross-culturally, this pattern holds: M. Liu and Lei (2005) confirmed that Agreeableness predicted perceived social support among Chinese adolescents, though cultural nuances (e.g., collectivistic emphasis on relational harmony) may amplify this effect. Together, these findings suggest that Agreeableness functions as a “social resource builder,” shaping how individuals perceive and engage with support networks, which in turn impacts their SWB.

Additionally, external family factors exert an influence on individual’s perceived social support. Family socioeconomic status (SES)—often operationalized via family income—emerges as a key environmental factor influencing both perceived social support and SWB. Research indicates that family income correlates with adolescents’ access to social support: higher-income families facilitate greater peer support and overall perceived support (Tsai, 2006; Salonna et al., 2012; H. Liu et al., 2024). Family SES also exerts multifaceted effects on psychological outcomes, including mental health, academic development, and emotion regulation (Li et al., 2013; Long & Pang, 2016; Xu et al., 2021; Wu et al., 2024), with some studies documenting its mediating or moderating role in the pathways linking social support to well-being (Guo & Li, 2025; Chu et al., 2019).

Despite the cumulative evidence on these individual constructs, three critical research gaps remain. First, the inconsistent Agreeableness-SWB relationship lacks a theoretical framework to reconcile divergent findings, particularly in collectivistic cultural contexts, like China. Second, few studies have integrated Agreeableness, perceived social support, and family SES into a unified model to examine their interactive effects on SWB. Third, the mediating role of perceived social support in the Agreeableness-SWB link, and how this mediation is moderated by family SES, has not been systematically explored.

Against this backdrop, the present study aims to: (1) clarify the direct association between Agreeableness and SWB among Chinese college students; (2) test the mediating role of perceived social support in this relationship; and (3) examine the moderating effect of family SES mediating pathway. By addressing these aims, this study contributes to the literature by providing a nuanced, integrative model of SWB antecedents and offering practical implications for promoting adolescent well-being in Chinese culture settings

## 2. Theoretical Background

This section elaborates on three core theories that underpin the research model: Social Exchange Theory, Emotional Regulation Theory, and the Family Stress model. These frameworks guide the logical derivation of hypotheses regarding the relationships among Agreeableness, perceived social support, family SES, and SWB.

### 2.1. Social Exchange Theory

Social exchange theory (Blau, 1964) posits that social interactions are governed by the principle of reciprocal give-and-take, where individuals seek to maximize rewards (e.g., emotional support, trust) and minimize costs (e.g., conflict, betrayal). Central to this theory is the proposition that relationship maintenance depends on perceived equity: when interactions are mutually beneficial, individuals are motivated to sustain and deepen social bonds.

Agreeableness aligns closely with the core tenets of social exchange theory. Individuals high in Agreeableness are predisposed to engage in prosocial behaviors (e.g., helping, cooperating, empathizing), which constitute “rewards” in social interactions (John & Srivastava, 1999). These behaviors elicit reciprocal positive responses from others, such as trust, support, and approval, thereby enhancing the agreeable individual’s perceived social support (S. J. T. Branje et al., 2005). In turn, the receipt of social support fulfills fundamental psychological needs for belongingness (Baumeister & Leary, 1995), which contributes to higher SWB. Conversely, individuals low in Agreeableness may exhibit more competitive or antagonistic behaviors, leading to strained social interactions, reduced social support, and lower SWB. From this theoretical perspective, perceived social support is hypothesized to mediate the relationship between Agreeableness and SWB.

### 2.2. Emotional Regulation Theory

Emotional regulation theory (Gross, 2002) defines emotional regulation as the processes by which individuals influence which emotions they have, when they have them, and how they experience and express these emotions. A core proposition of the theory is that adaptive emotional regulation strategies (e.g., cognitive reappraisal) enhance psychological well-being, whereas maladaptive strategies (e.g., expressive suppression) are linked to negative outcomes such as anxiety and depression.

Agreeableness is associated with adaptive emotional regulation capacities, which may explain its potential link to SWB. Research indicates that agreeable individuals are more likely to use cognitive reappraisal to manage negative emotions: they tend to reframe interpersonal conflicts in positive terms and prioritize empathy over retaliation (Graziano et al., 2007). This adaptive regulation reduces the frequency and intensity of negative affect, directly contributing to higher SWB. Additionally, agreeable individuals’ ability to regulate emotions facilitates positive social interactions, as they are better equipped to navigate interpersonal tensions and maintain supportive relationships. This, in turn, increases their perceived social support, creating an indirect pathway through which Agreeableness influences SWB. Thus, emotional regulation theory supports the mediating role of perceived social support by highlighting how Agreeableness shapes both emotional experiences and social resource acquisition.

### 2.3. The Family Stress Model

The family stress model (Masarik & Conger, 2017) provides a foundational framework for understanding how family socioeconomic status (SES) shapes adolescent development through stress transmission pathways. The core proposition of this theory is that economic hardship (low family SES) elevates parental psychological distress and interparental conflict, which in turn disrupts warm, supportive parenting practices and erodes family functioning. Over time, this chronic stress environment impairs adolescents’ social-emotional adjustment and reduces their access to adaptive coping resources—including the ability to establish and maintain supportive social relationships.

To fully explain the moderating role of family SES in the Agreeableness–perceived social support–SWB pathway, the family stress model is complemented by the resource compensation perspective (Hobfoll, 2001), a classic framework in psychology for understanding how resource availability interacts with individual traits to shape outcomes. This perspective posits that higher family SES provides adolescents with both tangible resources (e.g., opportunities for extracurricular participation, access to mentorship programs) and intangible resources (e.g., parental social capital, cultural capital), which can amplify the positive effects of individual traits and compensate for potential deficits in social support acquisition.

Integrating these two frameworks, we can delineate the moderating mechanism: For adolescents from high-SES families, abundant family resources can reinforce the positive link between Agreeableness and perceived social support. Agreeable traits facilitate prosocial interactions, and family resources further expand their social networks (e.g., through organized social activities) and enhance the visibility of their prosocial behaviors, thereby strengthening the conversion of Agreeableness into actual social support. This amplified social support then more effectively promotes SWB. In contrast, adolescents from low-SES families are embedded in the stress transmission pathway outlined by the family stress model: economic hardship may lead to family conflict, reduced parental support, and limited social opportunities. Even if they possess high Agreeableness, these contextual constraints may hinder their ability to translate prosocial tendencies into sufficient social support—attenuating the indirect effect of Agreeableness on SWB through perceived social support.

## 3. Hypotheses

The present study is a cross-sectional investigation designed to systematically delineate the complex interplay among Agreeableness, perceived social support, family income, and SWB. Based on the abovementioned theoretical framework of Agreeableness, perceived social support, subjective well-being, and SES. the following hypotheses proposed: H1: Agreeableness positively correlates subjective well-being (SWB); H2: Perceived social support plays a mediating role in the relationship between Agreeableness and SWB; H3: Family yearly income significantly moderates the indirect effect of Agreeableness on SWB through perceived social support, with this moderation operating exclusively on the first stage of the mediating pathway.

## 4. Methods

### 4.1. Participants

This study targeted college students as research participants and employed a cluster random sampling method. Questionnaires were distributed online via the Internet platform (a professional online survey tool in China) to over 100 universities across China. A total of 3682 questionnaires were collected; after excluding invalid questionnaires (e.g., those with incomplete submissions, random answers, systematic polarized responding and indications of social desirability bias), 3206 valid questionnaires remained for final analysis. Among the valid participants, there were 1812 males and 1394 females; in terms of academic year, 1507 were freshmen, 751 were sophomores, 713 were juniors, and 235 were seniors or above (e.g., postgraduate students enrolled in undergraduate-integrated postgraduate programs).

### 4.2. Measurements

#### 4.2.1. Personality Trait

The Agreeableness subscale of the Chinese Big Five Personality Inventory-Short Version (CBF-PI-B), revised by Wang et al. (2011), was used in this study. This questionnaire adopts a 6-point Likert scale and consists of 8 items. In previous studies, the Cronbach’s α coefficient of the Agreeableness subscale (Big Five Inventory) was 0.73–0.79 (Rammstedt & John, 2007; Ashton & Lee, 2007). In the present study, the Cronbach’s α coefficient for this subscale was 0.811.

#### 4.2.2. Subjective Well-Being

The Index of Well-Being (IWB), developed by Campbell in 1976, comprises two components: the General Affect Index and the Life Satisfaction Scale. The General Affect Index consists of 8 items, which describe the connotation of affect from different perspectives; the Life Satisfaction Scale contains only 1 item and uses a 7-point scoring system. When calculating the total score, the average score of the General Affect Index is added to the score of the Life Satisfaction Scale (with a weight of 1.1). The possible range of the total score is from 2.1 (representing the lowest level of well-being) to 14.7 (representing the highest level of well-being). For this scale, the Cronbach’s α coefficient of the General Affect Index is 0.85 (X. Zhang & Zheng, 2005). In the present study, the Cronbach’s α coefficient was 0.962.

#### 4.2.3. Perceived Social Support

The Chinese version of the Multidimensional Scale of Perceived Social Support (MSPSS), originally developed by Zimet in 1988, was used in this study. In 2001, Jiang Qianjin, a Chinese scholar, revised this scale and developed the Chinese-version Perceived Social Support Scale (PSSS), which includes two dimensions: intra-family support and extra-family support, with a total of 12 items. The scale uses a 7-point Likert scoring system, ranging from 1 (“strongly disagree”) to 7 (“strongly agree”); a higher total score indicates a higher level of perceived social support. Specifically, items 3, 4, 8, and 11 measure family support; items 6, 7, 9, and 12 measure friend support; and items 1, 2, 5, and 10 measure support from others. Total scores are categorized into three levels: low support (12–36 points), moderate support (37–60 points), and high support (61–84 points). For this scale, the overall Cronbach’s α coefficient is 0.921 (Jiang, 2001). In the present study, the Cronbach’s α coefficient for the total scale was 0.957.

#### 4.2.4. Family Income

With reference to the household monthly income classification in the study by Xie and Lv (2023), the income variable in this study was defined as family yearly income, which was categorized into 5 levels: “10,000–50,000 RMB”, “51,000–100,000 RMB”, “101,000–150,000 RMB”, “151,000–200,000 RMB”, and “above 200,000 RMB”. These levels were assigned numerical values from 1 to 5, respectively, where a higher value indicates a higher family yearly income.

### 4.3. Statistical Analysis

Data were collected through online platforms. Following data collection, invalid responses were excluded based on predetermined criteria, including incomplete submissions, uniform responses across all items, systematic polarized responding, and indications of social desirability bias. Systematic polarized responding is defined as responses that consistently choosing the endpoints of Likert scale items (e.g., repeatedly selecting 1 or 7 in a 7-point scale) with no logical consistency in responses. This determination is made by two independent researchers. According to the Marlowe-Crowne Social Desirability Scale (short version), responses with scores above the 80th percentile in the sample distribution were classified as having social desirability bias (Crowne & Marlowe, 1960; Strahan & Gerbasi, 1972). In this study, the missing date for all variables was low. Therefore, following general recommendations for cross-sectional studies was small missing data proportions, we used listwise deletion (van der Ark & Sijtsma, 2005).

Statistical analyses were performed using SPSS 25.0 with the PROCESS v3.5 extension to test the hypothesized relationships. The analytical procedure consisted of three sequential steps: First, common method bias was assessed through exploratory factor analysis to determine whether significant variance could be attributed to the measurement method. Second, the direct association of Agreeableness with subjective well-being was examined. Subsequently, provided that a significant direct association was established in the previous step, the mediating role of perceived social support in the relationship between Agreeableness and subjective well-being was investigated. And last, based on the preceding steps, the moderating effect of family income on the relationship between Agreeableness and perceived social support was tested.

### 4.4. Control and Test of Common Method Bias

All scales used in this study are self-reported scales, and the data are derived from participants’ self-perceptions. Participants often exhibit biases in their responses due to factors such as subjective tendencies and social desirability. Therefore, in this study, in addition to controlling for bias during the questionnaire administration process (e.g., designing different scoring methods, anonymous survey administration and varying questionnaire prompts), Harman’s single-factor test was employed to examine common method bias.

Specifically, an exploratory factor analysis (EFA) was conducted on the raw data of the main study variables. The results showed that there were 5 factors with eigenvalues greater than 1, and the variance explained by the first factor was only 30%, which was lower than the critical threshold of 40%. This indicates that there was no significant common method bias in this study.

## 5. Results

### 5.1. Descriptive Statistics Among Variables

The study examined three latent variables: Agreeableness, perceived social support, and Subjective Well-being (Index of Well-being). The means, standard deviations, and Pearson correlation coefficients for the measured variables are summarized in Table 1. Agreeableness, perceived social support, and SWB were significantly correlated with each other. The pattern of correlation coefficients suggests the potential presence of mediating effects among these variables. Given that gender showed significant correlations with Agreeableness, the Index of Well-being, and perceived social support, and that annual household income also demonstrated statistically significant relationships with these three variables, both gender and annual household income were included as control variables in subsequent analyses.

### 5.2. Test of the Direct Association Between Agreeableness and Subjective Well-Being

In this study, gender and family yearly income were included as control variables. A hierarchical regression analysis was conducted to examine the predictive association between Agreeableness and SWB. Gender and family yearly income were entered in the first step of the regression model, followed by Agreeableness in the second step.

The results of the hierarchical linear regression analysis, as detailed in Table 2, indicated that after controlling for gender and family yearly income, Agreeableness remained significantly associated with SWB (β = 0.239, t = 13.786, *p* < 0.001). Thus, hypothesis H1 was supported.

### 5.3. Test of the Mediation Model

The mediation effect of perceived social support in the relationship between Agreeableness and subjective well-being was examined using a stepwise regression approach, controlling for gender and annual household income. The results are presented in Table 3. The total effect of Agreeableness on SWB was significant (β = 0.928, t = 13.786, *p* < 0.001). After introducing perceived social support as a mediator, the direct association between Agreeableness and SWB remained significant (β = 0.503, t = 8.142, *p* < 0.001). Agreeableness demonstrated a significant positive association with perceived social support (β = 0.425, t = 12.789, *p* < 0.01). Furthermore, perceived social support was a significant positive contributor to SWB (β = 0.522, t = 34.501, *p* < 0.001).

**Figure 1:**
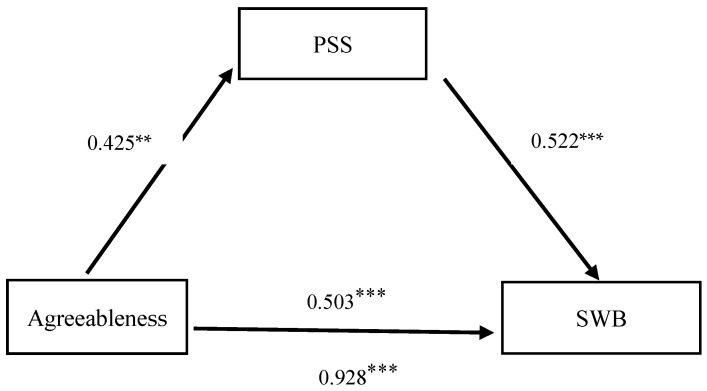
Path Diagram of the Mediation Model.

Besides, Bootstrap test results showed that the direct effect value of Agreeableness on SWB was 0.503, with a corresponding 95% confidence interval (CI) of [0.377, 0.615]; the mediating effect value of perceived social support in the relationship between Agreeableness and SWB was 0.425, with a corresponding 95% CI of [0.374, 0.559]. The results are presented in Table 4 and Figure 1. Neither of these intervals contained zero, thus supporting the original hypotheses: Agreeableness not only directly associates with SWB but also correlates to SWB indirectly through the mediating role of Perceived Social Support.

Further analysis revealed that the mediating effect (0.425) accounted for 46% of the total effect (0.928), while the direct effect (0.503) accounted for 54% of the total effect (0.928). These results confirm that the mediating role of perceived social support in the model is valid and belongs to a partial mediation effect. Thus, hypothesis H2 was supported.

### 5.4. Test of the Moderated Model

Furthermore, Model 7 of the PROCESS macro was employed to examine the moderating effect, controlling for gender. The results presented in Table 5 indicate that after including family yearly income in the model, the interaction term between Agreeableness and family yearly income significantly associated with perceived social support (β = 0.143, t = 6.397, *p* < 0.001). This result suggests that family yearly income positively associates with perceived social support and serves as a positive moderator in the relationship between Agreeableness and perceived social support.

**Figure 2:**
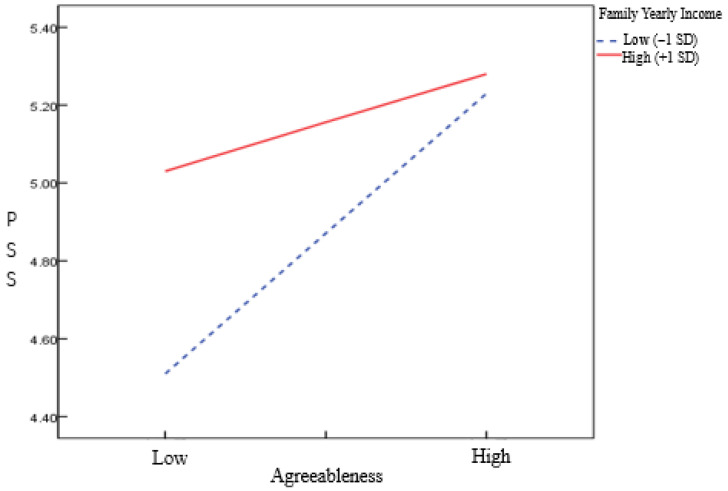
Moderating Effect of PSS in the Agreeableness-SWB Relationship.

As illustrated in the simple slope plot (Figure 2), for the group with lower family yearly income (M − 1 SD), Agreeableness had a significant positive link to perceived social support. Similarly, for the group with higher annual household income (M + 1 SD), Agreeableness also demonstrated a positive association with perceived social support, though the magnitude of this effect was smaller compared to the lower income group. These results indicate that, the positive influence of Agreeableness on perceived social support is reduced as income increases. Specifically, the effect of Agreeableness on perceived social support is stronger under conditions of lower family yearly income compared to higher family yearly income. Thus, hypothesis H3 was supported.

### 5.5. Moderated Mediation Effect

To test whether family yearly income moderates the indirect effect of Agreeableness on SWB via perceived social support (a moderated mediation model), we used PROCESS Model 7 with gender controlled. Model 7 was selected as it is tailored for first-stage moderation (i.e., moderator acts on Agreeableness → perceived social support), aligning with our hypothesis.

The analysis presented in Table 6 revealed a significant index of moderated mediation (index = 0.075, 95% CI [0.032, 0.121]), confirming the existence of a moderated mediation effect. This finding indicates the indirect effect varies with family yearly income, operating solely through the first stage of the mediation pathway.

Probing conditional indirect effects at M ± 1 SD of family yearly income revealed that the indirect effect was stronger in the lower-income group (effect = 0.132, SE = 0.018, 95% CI [0.098, 0.169]) and attenuated in the higher-income group (effect = 0.057, SE = 0.012, 95% CI [0.035, 0.082]). This difference derives from family income’s moderation on the Agreeableness → perceived social support link. These findings confirm the proposed moderated mediation model: family yearly income reduces the strength of the mediating pathway via perceived social support, making Agreeableness’s indirect effect on SWB more pronounced in lower-income contexts. Consistency with prior moderation results supports the model’s robustness. In summary, the moderated mediation model is fully supported. Family yearly income significantly moderates the indirect effect of Agreeableness on SWB through perceived social support (first-stage moderation), validating Hypothesis H3.

## 6. Discussion

### 6.1. Association Between Agreeableness and SWB

In the present study, Agreeableness was significantly and positively correlated with SWB, which is consistent with the findings of previous studies (Anglim et al., 2020; Z. Zheng et al., 2023). Traditional personality research suggests that individuals with high Agreeableness tend to display more prosocial behaviors, such as caring for others and being tolerant. These behaviors typically help them establish harmonious interpersonal relationships, which in turn enhance their life satisfaction and affective well-being (Diener et al., 2009). Consequently, individuals with high Agreeableness often report higher levels of life satisfaction and positive affect. A longitudinal study conducted on Chinese adults found that agreeableness significantly improved life satisfaction by strengthening family support and the quality of friendships (R. Zhang & Li, 2017).

Studies have indicated that Agreeableness exerts an instrumental effect on well-being, specifically, it influences individuals’ well-being by selecting and creating environments and life events that promote happiness (McCrae & Costa, 1991). Additionally, individuals with high Agreeableness can control the spread of affect activation related to hostility through self-regulation processes, thereby making them less likely to experience negative affect (Gross & Thompson, 2007).

Notably, the positive association between Agreeableness and SWB observed in this study stands in contrast to some previous research that reported a negative or non-significant correlation between the two variables (Headey & Wearing, 1989; Lucas & Fujita, 2000). We argue that the divergence in findings may stem from two factors. First, contextual differences in cultural backgrounds may play a critical role. Most studies reporting negative correlations were conducted in individualistic Western cultures, where excessive Agreeableness may be perceived as a violation of personal autonomy and individual goals, thereby undermining SWB. In contrast, the present study was conducted in a Chinese collectivistic cultural context, where prosocial behaviors and interpersonal harmony—core characteristics of Agreeableness—are highly valued and rewarded by society (Bond, 1988). This cultural emphasis may amplify the positive impact of Agreeableness on SWB.

Second, differences in the operationalization and measurement of Agreeableness and SWB could contribute to the contradictions. Some studies that found negative correlations used narrow measures of Agreeableness that overemphasized submissiveness and passivity (Costa & McCrae, 1992), while the present study adopted a comprehensive measure that includes prosociality, empathy, and interpersonal warmth—dimensions that are more closely aligned with positive social outcomes in collectivistic contexts. Regarding SWB, studies focusing on the affective component may yield different results than those focusing on the cognitive component. For instance, Lucas and Fujita (2000) found that Agreeableness was negatively correlated with positive affect in competitive work environments, but not with life satisfaction.

In-depth exploration of the negative Agreeableness-SWB link in existing literature further reveals that such findings are often context-specific. For example, Headey and Wearing (1989) found that highly agreeable individuals were more likely to experience decreases in SWB when facing interpersonal conflicts, as they were less likely to assert their own needs and more prone to emotional distress. However, this pattern may not hold in contexts where conflicts are resolved through collective harmony, as in Chinese culture. Additionally, in competitive environments, such as high-pressure workplaces or academic settings, agreeable individuals may be less likely to prioritize their own interests, leading to lower SWB due to unmet personal goals (Judge et al., 2002). These findings highlight the importance of considering contextual factors when examining the Agreeableness-SWB relationship.

### 6.2. The Mediating Role of Perceived Social Support

Correlation analysis in this study revealed that pairwise correlations existed among Agreeableness, perceived social support, and SWB. This finding provides a basis for the subsequent test of the mediating role of perceived social support in the relationship between Agreeableness and SWB.

After conducting a Bootstrap-based mediation effect test, it was found that perceived social support played a mediating role in the relationship between Agreeableness and SWB, with the mediating effect accounting for 46% of the total effect. Meanwhile, the direct effect of Agreeableness on SWB accounted for 54% of the total effect. These results indicate that Agreeableness not only directly links to SWB but also associates SWB indirectly through perceived social support, which confirms a partial mediation effect. After controlling for the effects of gender and age, perceived social support demonstrated a significant mediating role in the relationship between Agreeableness and SWB. With gender and age statistically controlled, Agreeableness positively associated with perceived social support, indicating its unique contribution to the perception of social support. Generally, individuals with high levels of Agreeableness tend to be more caring, understanding, and compassionate. These characteristics facilitate the establishment of positive social connections and enhance their perception of available social support.

According to Social Exchange Theory, prosocial behaviors in interpersonal interactions can elicit reciprocal support from others. Highly agreeable individuals, who exhibit warmth and kindness, are more likely to receive positive responses from others, thereby fostering a beneficial cycle of social support. Furthermore, these individuals typically prefer conflict avoidance and employ constructive communication strategies, which help reduce interpersonal friction and tension, consequently increasing others’ willingness to provide support. Highly agreeable individuals often maintain higher-quality social relationships, building interpersonal networks characterized by greater trust and reliability. As a result, they tend to perceive social support more strongly. Social Support Theory emphasizes that the quality of supportive relationships is more critical than their quantity (Cohen & Wills, 1985), which further explains why Agreeableness can positively predict perceived social support.

From the perspective of Emotion Regulation Theory, highly agreeable individuals generally exhibit better emotional regulation abilities, enabling them to maintain positive affective states—a key factor in sustaining high levels of subjective well-being. Improved emotion regulation capacity also helps mitigate the erosive impact of negative emotions on well-being (Gross, 2002). This result indicates that Agreeableness positively associates with SWB. Meanwhile, Agreeableness can also indirectly influence SWB through perceived social support: individuals with higher levels of Agreeableness tend to have higher perceived social support, which in turn leads to higher levels of SWB.

Additionally, support is a process of mutual exchange. Those who provide support also strengthen their own altruism and social identity, forming a positive cycle. Precisely because of their higher level of empathy, individuals with high Agreeableness are better able to make others feel their kindness and support. In response, others also express support and kindness, which enables these individuals to perceive more social support, build higher-quality interpersonal interactions, and ultimately improve their SWB.

### 6.3. Linking the Current Findings to Autobiographical Memory

Consistent with the thematic focus of this Special Issue on autobiographical memory, the mediating mechanism of perceived social support in the relationship between Agreeableness and SWB identified in the current study is inherently rooted in autobiographical memory processes. As noted, social support is inherently tied to interpersonal interactions in individuals’ lived environments, and any individual’s description or evaluation of such supportive interactions necessarily involves reflecting on and retrieving events from their autobiographical memory. Specifically, when individuals with high Agreeableness perceive social support, they are essentially engaging in autobiographical recall of past prosocial exchanges, care experiences, or harmonious interpersonal events—memories that shape their current appraisal of available social resources. This connection is supported by existing empirical research on autobiographical memory and social support. Another line of study has shown that writing autobiographical narratives about supportive interpersonal experiences facilitates the integration of these memories into one’s self-concept, thereby strengthening the enduring perception of social connectedness and improving psychological well-being (Pennebaker & Seagal, 1999). In this sense, the healthy coping mechanism (i.e., perceiving and utilizing social support) that bridges Agreeableness and SWB in our model is not only a social cognitive process but also a product of autobiographical memory activation. High Agreeableness may facilitate the encoding and retrieval of positive social interaction memories: individuals with this trait are more likely to attend to and retain memories of supportive interpersonal experiences, which in turn strengthens their perceived social support and ultimately enhances SWB. This connection underscores that autobiographical memory serves as a foundational cognitive substrate for the observed mediating effect, linking the current study’s findings to the broader literature on autobiographical memory and well-being.

### 6.4. The Moderating Role of Family Yearly Income

Based on the mediation model, this study further tested the moderating role of family yearly income. The result of test revealed that family yearly income exerted a significant moderated mediation effect on the interplay between Agreeableness, perceived social support and SWB, as evidenced by a statistically significant moderated mediation (index = 0.075, 95% CI [0.032, 0.121]) with the 95% bias-corrected bootstrap confidence interval excluding zero. Family income exerts a moderating effect exclusively at the first stage of the mediation chain, a critical mechanism that underpins the presence of the moderated mediation effect in this study. Unlike conventional moderating effects that merely alter the magnitude of a direct bivariate relationship, the moderated mediation effect identified herein reflects a more nuanced conditional process: family income does not directly modulate the link between perceived social support and SWB; instead, it regulates the efficiency with which Agreeableness translates into perceived social support, thereby indirectly shaping the overall indirect effect of Agreeableness on SWB. Probing of the conditional indirect effects at the mean ±1 SD of family income revealed that the indirect effect of Agreeableness on SWB via perceived social support was markedly stronger among those individuals from lower income households (effect = 0.132, CI [0.098, 0.169]) and notably attenuated among those from higher income backgrounds (effect = 0.057, CI [0.035, 0.082]).

This finding underscores a pivotal theoretical assertion and this statistically significant moderated mediation index further corroborates the reliability of the observed between-group disparity in indirect effects to substantiate this claim. Specifically, Agreeableness appears to be more susceptible to the influence of lower household income levels in shaping perceived social support, a finding consistent with previous research (Kothgassner et al., 2019; Tsai, 2006; Woodward & Fergusson, 1999). A parsimonious interpretation of this pattern is that lower-income individuals are more reliant on social resources derived from prosocial personality traits to mitigate the adverse psychological impacts of material deprivation, whereas their higher-income counterparts can leverage alternative financial resources to compensate for the well-being benefits conferred by social support. When household income is relatively low, increases in Agreeableness are associated with a more pronounced upward trend in perceived social support. This result suggests that in resource-scarce environments, an individual’s Agreeableness plays a substantial role in enhancing their perception of available social support. Conversely, among individuals with higher family yearly income, the relationship between Agreeableness and perceived social support is flatter. This pattern implies that individuals from higher-income backgrounds tend to perceive consistently high levels of social support regardless of their inherent Agreeableness levels.

According to Xie and Lv (2023), socioeconomic status influences an individual’s support systems and social networks. This phenomenon can be explained through the lens of social exclusion theory: since the cost of maintaining extensive social networks often outweighs the supportive resources they provide, individuals with lower socioeconomic status are frequently excluded from such networks (Vrooman & Hoff, 2013; Brown et al., 2020). The researchers argued that individuals in socioeconomically disadvantaged positions typically possess smaller social networks and consequently receive less social support. The current study further demonstrates that the influence of Agreeableness on perceived social support is moderated by family yearly income, with this effect being more pronounced among university students from lower-income families. In low-income environments, the prosocial behaviors exhibited by agreeable individuals (such as proactively helping others) are more readily recognized by their community and reciprocated with support, thereby establishing a “complementary compensation mechanism” that amplifies their perception of social support.

The findings may also be explained from the perspectives of evolution and social interaction theory: Differences in resource distribution among human groups lead to distinct social cognitive patterns (X. Zheng et al., 2015). Individuals in the lower social strata, due to limited resources and exposure to survival threats, often rely on others and groups for survival, thus developing a group-oriented and other-oriented mindset. In contrast, individuals in the upper social strata, with abundant resources, tend to be more egocentric. This outcome is jointly driven by objective survival needs and long-term evolutionary processes. Individuals with high household income often have abundant material resources, which relatively weakens the role of Agreeableness. This outcome can be attributed to the fact that material resource exchange is characterized by greater convenience and efficiency, while avoiding the excessive depletion of emotional resources. Conversely, for individuals with low household income, demonstrating Agreeableness incurs relatively low costs but yields higher benefits, such as assistance and trust from others, thereby enhancing their perception of social support.

Therefore, family yearly income moderates the relationship between Agreeableness and perceived social support. As perceived social support mediated the effect of Agreeableness on SWB, the moderated mediation effect showed that the effect of Agreeableness on SWB through perceived social support was stronger for low-income individuals and attenuated for high-income individuals. Specifically, higher Agreeableness is associated a marked increase in perceived social support among low-income groups, whereas this positive link is substantially attenuated in high-income groups.

### 6.5. Theoretical and Practical Implications

Our findings make three key theoretical contributions, which address critical gaps in existing literature and advance relevant theoretical frameworks, that deepen the understanding of the interplay between personality, socioeconomic factors, and well-being. First, we enrich the cross-cultural application of Emotional Regulation Theory by demonstrating that Agreeableness serves as a culture-specific emotional regulation resource enhancing SWB in Chinese collectivistic contexts. Specifically, in a cultural environment that values interpersonal harmony and group cohesion, the prosocial tendencies inherent in Agreeableness enable individuals to effectively regulate emotional states through positive social interactions, which extends the theory’s contextual adaptability beyond Western individualistic settings.

Second, we advance Social Support Theory by identifying family annual income as a critical boundary condition for the personality-perceived social support link. This finding fills important gaps in existing research that has predominantly focused on+ social network structure and support quality while neglecting the modulating role of socioeconomic factors in how personality traits translate into perceived social support. Traditional Social Support Theory emphasizes the role of social networks and support quality in facilitating support perception and utilization (Cohen & Wills, 1985), but it rarely considers how socioeconomic factors influence the extent to which personality traits shape support perception. For instance, prior studies have established a positive association between Agreeableness and perceived social support, yet they fail to address whether this association varies across different socioeconomic groups. Our findings fill this void by revealing that the predictive power of Agreeableness on perceived social support varies with family income: the positive effect of Agreeableness on perceived social support is more pronounced among individuals with higher family income, whereas this effect is weakened among those with lower family income. This suggests that socioeconomic context modulates the conversion of personality resources (i.e., Agreeableness) into social support resources, addressing a key gap in prior research on the boundary conditions of social support formation.

Third, we reconcile contradictory findings on the Agreeableness-SWB relationship by clarifying the indirect, context-dependent mechanism. Specifically, Agreeableness influences SWB through perceived social support, and this mediating process is further moderated by family income—thus providing a more nuanced theoretical explanation for the divergent results. Some studies have reported a strong positive Agreeableness-SWB link, while others have found a weak or non-significant association. Our moderated mediation model demonstrates that this relationship is indirect (operated through perceived social support) and context-dependent (moderated by family income), providing a unified framework to reconcile these conflicting results and advancing the theoretical understanding of the complex pathways linking personality to well-being.

Based on our findings, we propose targeted, actionable practical recommendations tailored to different stakeholders to maximize the translation of theoretical insights into real-world interventions. For educators, given that the positive effect of Agreeableness on perceived social support is more pronounced among low-income students, efforts should focus on fostering Agreeableness and interpersonal skills (e.g., through cooperative learning activities, empathy training programs) among this group. Additionally, creating prosocial-valuing school environments (e.g., establishing peer support systems, recognizing prosocial behaviors) can further strengthen their perceived social support and ultimately enhance SWB. For mental health professionals, interventions should be stratified by family income levels: for low-income agreeable individuals, interventions should prioritize enhancing social networks (e.g., connecting them with community support groups, providing social skill-building workshops) to capitalize on the strong positive link between their Agreeableness and perceived social support; for high-income groups, interventions should aim to shift focus to deepening the quality of existing social connections rather than expanding network size. For policymakers, developing comprehensive community support systems for low-income families (e.g., affordable counseling services, community-based mutual aid programs, economic assistance initiatives) is crucial. These systems can amplify the strong positive association between Agreeableness and social support among low-income families. This pathway is far more pronounced in this group than in higher-income households, thereby mitigating the adverse impacts of socioeconomic disadvantages on their SWB.

### 6.6. Limitations and Future Prospects

Despite its contributions, the present study has several limitations that should be acknowledged, and corresponding directions for future research are proposed to address these limitations below. First, the cross-sectional research design precludes causal inferences. Although we propose a moderated mediation model where Agreeableness influences subjective well-being (SWB) through perceived social support, with family income as a moderator, the cross-sectional data cannot confirm the direction of causality. For example, it is possible that higher SWB leads to more prosocial behaviors (higher agreeableness) and enhanced perceived social support, rather than the other way around. To rectify this limitation, future research should conduct longitudinal studies to track the temporal changes in the variables and verify the causal relationships in the proposed moderated mediation model. Second, the data were collected through self-report questionnaires, which may introduce response bias; participants may have overreported their Agreeableness, perceived social support, and SWB to conform to social expectations. To mitigate such bias, future research should integrate multiple sources of data, such as peer reports and objective measures of social support (e.g., frequency of actual social support received), to triangulate the study findings.

Third, the sample may lack generalizability. The present study focused on university students, who may have different characteristics than non-student young adults, and the sample was collected from a single region in China, further limiting the generalizability of the findings to other cultural and geographic contexts. To enhance the external validity of the results, future research should include more diverse samples, such as non-student populations and individuals from different regions and cultural backgrounds, and undertake cross-cultural and regional (e.g., urban vs. rural) comparative studies. Fourth, we only examined family yearly income as a moderator, while other potential moderating variables (e.g., region, gender, age, and other personality traits) may also influence the relationships among the variables. For instance, gender differences in the expression of Agreeableness and perception of social support may alter the moderated mediation effect. To refine the proposed model, future research should explore additional moderators and mediators (e.g., religious factors, coping styles) and systematically test their roles in the relationships between Agreeableness, perceived social support, and SWB. Additionally, adopting mixed-methods designs that combine quantitative and qualitative approaches could provide in-depth insights into the underlying mechanisms of these relationships.

### 6.7. Conclusions

This study investigates the relationship between Agreeableness and SWB, examining the mediating role of perceived social support and the moderating role of family annual income. The results confirm that Agreeableness is positively associated with SWB, and this relationship is partially mediated by perceived social support—notably, individuals need to invoke autobiographical memory to perceive and process such social support. Furthermore, family annual income moderates the mediating effect by regulating the relationship between Agreeableness and perceived social support, with the effect being more pronounced in low-income groups.

These findings resolve contradictory results in the literature by highlighting the contextual nature of the Agreeableness-SWB relationship and clarifying the underlying mechanism, which incorporates the role of autobiographical memory in social support perception. The theoretical implications extend existing theories on emotional regulation, social support, and personality and well-being. The practical recommendations provide actionable strategies for enhancing SWB among different socioeconomic groups. Despite its limitations, this study contributes to a deeper understanding of the factors influencing SWB and offers valuable insights for future research and practice. Ultimately, the findings emphasize the importance of considering both individual personality traits, the role of autobiographical memory in social cognition, and socioeconomic context in promoting subjective well-being.

## Figures and Tables

**Table 1:** Descriptive Statistical Results and Correlation Coefficients of Variables.

	M ± SD	1	2	3
1 Agreeableness	2.582 ± 0.656	1.00		
2 Index of Well-Being	10.511 ± 2.640	0.233 **	1.00	
3 Perceived social support	5.026 ± 1.123	0.193 **	0.540 **	1.00
4 Genger		0.130 **	−0.052 **	0.098 **
5 Age		0.019	0.012	0.001
6 Family yearly income		0.104 **	0.66 **	0.109 **

Note: ** The correlation was significant at the 0.01 level (two-tailed).

**Table 2:** Results of Hierarchical Regression Analysis for the Association Between Agreeableness and Subjective Well-Being.

Variables	Layer 1		Layer 2	
	β	t	β	t
Agender	−0.059	−3.320	−0.030	−1.715
Family yearly income	0.071	4.020	0.093	5.402
Agreeableness			0.239	13.786 ***
F	12.434 ***		72.130 ***	
R^2^	0.008		0.063	
△R^2^	0.007		0.062	

Note: *** *p* < 0.001, ** *p* < 0.01, * *p* < 0.05. The same notation applies to the following figures and tables.

**Table 3:** Testing Mediation Effects via Stepwise Regression.

	Model 1		Model 2		Model 3	
	SWB		PSS		SWB	
	β	t	β	t	β	t
Agender	−0.030	−1.715	0.115	6.674 ***	−0.090	−6.036 ***
FYI	0.093	5.402 ***	0.121	7.029 ***	0.030	2.025 *
Agreeableness	0.928	13.786 ***	0.425	12.789 **	0.503	8.142 ***
PSS					0.522	34.501 ***
F	72.130 ***		25.5085 ***		371.762 ***	
R^2^	0.063		0.067		0.317	
△R^2^	0.056		0.066		0.316	

NOTE: SWB = Subjective Well-Being; PSS = Perceived Social Support; FYI = Family Yearly Income.

**Table 4:** Decomposition of Total, Direct, and Indirect Effects.

	Effect	Boot SE	Boot LLCI	Boot ULCI	Relative Effect
Total effect *c*	0.928	0.070	0.822	1.095	
Direct effect *c*’	0.503	0.061	0.377	0.615	54%
Indirect effect *ab*	0.425	0.047	0.374	0.559	46%

**Table 5:** Test of the Moderated Mediation Model.

		Coefficient Significance		Fit Indices		
Outcome Variable	Independent Variable	β	t	F	R	R^2^
PSS	Agreeableness	0.392	13.298 ***	67.659 ***	0.0279	0.078
	Family Yearly Income	0.105	6.886 ***			
	Agreeableness × Family Yearly Income	0.143	6.397 ***			
	Agender	0.249	6.382 ***			

**Table 6:** Decomposition of Effects in the Moderated Mediation Model.

	Effect Value	Boot SE	Boot LLCI	Boot ULCI	Relative Effect
Total effect *c*	0.928	0.070	0.822	1.095	
Direct effect *c*’	0.503	0.061	0.377	0.615	54%
Indirect effect *ab*	0.425	0.047	0.374	0.559	46%
Conditonal indirect effect (low, −1 SD)	0.132	0.018	0.098	0.169	14.2%
Conditional indirect effect (medium, SD)	0.425	0.047	0.374	0.559	46%
Conditional indirect effect (high, +1 SD)	0.057	0.012	0.035	0.082	6.1%
Index of moderated mediation	0.075		0.032	0.121	

## Data Availability

The original contributions presented in this study are included in the article. Further inquiries can be directed to the corresponding author.
